# Correction: Feng et al. Nicotinamide Phosphoribosyltransferase (Nampt)/Nicotinamide Adenine Dinucleotide (NAD) Axis Suppresses Atrial Fibrillation by Modulating the Calcium Handling Pathway. *Int. J. Mol. Sci.* 2020, *21*, 4655

**DOI:** 10.3390/ijms221910881

**Published:** 2021-10-08

**Authors:** Duo Feng, DongZhu Xu, Nobuyuki Murakoshi, Kazuko Tajiri, Rujie Qin, Saori Yonebayashi, Yuta Okabe, Siqi Li, Zixun Yuan, Kazutaka Aonuma, Masaki Ieda

**Affiliations:** Department of Cardiology, Institute of Clinical Medicine, Faculty of Medicine, University of Tsukuba, Tsukuba 305-8575, Japan; fengduoryu@outlook.com (D.F.); n.murakoshi@md.tsukuba.ac.jp (N.M.); ktajiri@md.tsukuba.ac.jp (K.T.); leopalace@sohu.com (R.Q.); syonebayashi789@gmail.com (S.Y.); yokabe0211@gmail.com (Y.O.); l.siqi@outlook.com (S.L.); YUAN1120364808@yahoo.com (Z.Y.); kaonuma@md.tsukuba.ac.jp (K.A.); mieda@md.tsukuba.ac.jp (M.I.)

The authors wish to make the following corrections to this paper [1]:

Changes in figures due to mislabeling. Figure 1E and Figure 4C were replaced with corrected figures. The picture changes will not affect the conclusion of the manuscript.

The authors would like to apologize for any inconvenience caused to the readers by these changes.

## Figures and Tables

**Figure 1:**
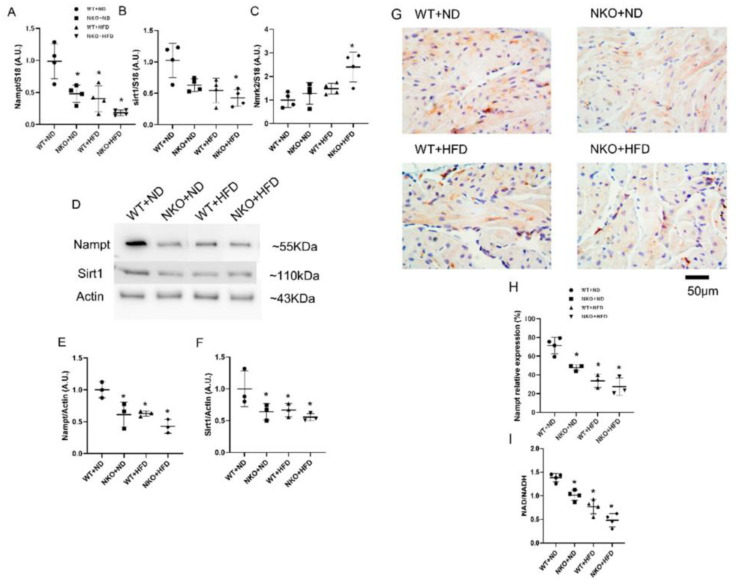
Gene and protein expressions of Nampt and Sirt1 in the atrial tissues. (**A**) Nampt mRNA expression levels in the atrial tissues evaluated by quantitative PCR (*n* = 4 mice per each group). (**B**) Sirt1 mRNA expression levels in the atrial tissues evaluated by quantitative PCR (*n* = 4 mice per group). (**C**) Nmrk2 mRNA expression levels in the atrial tissues evaluated by quantitative PCR (*n* = 4 mice per group). (**D**) Representative Western Blots for Nampt and Sirt1 in the atrial tissues (*n* = 3 mice per group). (**E**) Nampt protein expression levels in the atrial tissues (*n* = 3 mice per group). (**F**) Sirt1 protein expression levels in the atrial tissues (*n* = 3 mice per group). (**G**) Representative images of Nampt immunohistochemistry in the atrial tissues. Nampt was mainly expressed in the cytoplasm of atrial cardiomyocytes. Scale bar: 50 µm. (**H**) Positively stained areas in the atrial tissues by immunohistochemistry. (**I**) Atrial NAD/NADH ratio in four studied groups (*n* = 4 mice per group). * *p* < 0.05 vs. WT+ND mice. Data are shown as mean ± SD. Statistical comparisons between multiple groups: one-way ANOVA followed by a post hoc Bonferroni test.

**Figure 4:**
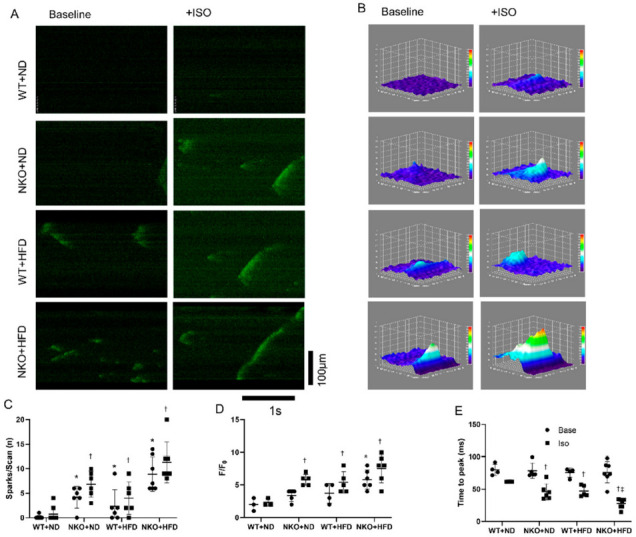
Calcium imaging of isolated cardiomyocytes. (**A**) Representative calcium images of isolated cardiomyocytes at baseline and after application of Iso obtained by confocal microscopy. (**B**) Representative images of 3D surface plot of calcium sparks of each groups. (**C**) Numbers of calcium sparks and mini waves per scan. Mini waves are defined as the calcium waves which did not transport throughout the cardiomyocyte, but only a portion of the cell. Base indicates baseline condition, and Iso indicates condition after adding isoproterenol (Iso). (**D**) Scatter chart of fractional fluorescence increases (F/F_0_). (**E**) Scatter chart of time to peak florescence signal. * p < 0.05 vs. WT+ND mice; ^†^ *p* < 0.05 vs. WT+ND+Iso mice; ^‡^ *p* < 0.05 vs. WT+HFD+Iso mice (*n* = 10 in WT+ND group, *n* = 7 in NKO+ND, *n* = 7 in WT+HFD group, *n* = 6 in NKO+HFD group). Statistical comparisons between multiple groups: one-way ANOVA followed by a post hoc Bonferroni test.
